# Centralization or decentralization? Power allocation in team innovation management

**DOI:** 10.1371/journal.pone.0310719

**Published:** 2024-10-28

**Authors:** Shiwen Luo, David Yoon Kin Tong

**Affiliations:** 1 School of International Business, Zhejiang Financial College, Hangzhou, China; 2 IUMW Business School, International University of Malaya-Wales, Jalan Tun Ismail, Malaysia; Fooyin University, TAIWAN

## Abstract

Power disparity, as an important form of internal team hierarchy, presents a "double-edged sword effect". To reconcile the inconsistent effects and systematically explore the different mechanisms of power disparity, this study constructs a comprehensive theoretical model based on power functionalism and power conflict theory, with team coordination and team conflict as dual mediators, and power legitimacy as moderator. By collecting valid questionnaires from 76 teams across 27 different types of companies in various regions, statistical analysis and hypothesis testing were conducted on the data. The results conclude that power disparity positively influences team innovation performance through the team coordination path and negatively affects it through the team conflict path. However, under the moderating effect of power legitimacy, the negative effect of the team conflict path is suppressed, and the positive effect of the team coordination path is strengthened, thus ensuring that power disparity has a positive effect on team innovation performance. This study provides a useful reference for designing the power hierarchy within enterprises, and offers profound insights into effective organizational structure and decision-making processes.

## 1 Introduction

Innovation is the foundation of enterprise development, and the key to enterprise innovation lies in team innovation. Previous studies on team innovation have predominantly been based on the assumption of a balanced power allocation [1, 2]. It is obvious that this assumption lacks ecological validity and fails to accurately depict the social relationships among team members, lagging behind modern organization management practice. Imbalanced power allocation in organizations is believed to be prevalent and not unique to top management teams (TMT) [3]. Power disparity, representing the degree of imbalance in power allocation [4], is widely defined as the asymmetric control of valuable resources [5]. In recent years, as power has become one of the most controversial concepts in the social, political, economic, and ethical domains [6], it has been widely recognized as a critical factor influencing organizational learning and success [7], the coordination of organizational information systems [8], and societal functioning. Consequently, power disparity has gradually received widespread attention. When power is concentrated in one member (centralization), the level of power disparity is at its highest, while when power is evenly allocated (decentralization), the level of power disparity is at its lowest. To encourage team innovation, should power allocation be centralized or decentralized? At present, there is no unified answer to this question.

Previous studies have presented two different views concerning the utility of power disparity. One is the constructive view of power functionalism, which believes that power disparity can benefit teams by enhancing role clarity among team members, promoting internal division of labor, unifying divergent opinions, improving interaction and coordination within the team [9–11]. The other is the destructive view of power conflict theory, which posits that power disparity can lead to power struggles within the team, trigger internal conflict, generate negative emotions such as perceived unfairness, and worsens interpersonal relationships, thereby harming the team [2, 12–14]. So, does power disparity lead to constructive or destructive outcomes for team innovation? Obviously, the inconsistent views highlight the need to construct a systematic theoretical model of the relationship between power disparity and team innovation that can integrate these two different mechanisms with the aim of comprehensively exploring the role of power allocation in the process of team innovation.

Power disparity does not directly affect team outcomes but influences them through its interaction with team processes [15]. However, current research on the process mechanisms of power disparity often focuses only on a single path and excludes other potential paths [12]. Obviously, this approach may exaggerate the importance of one of the relevant process mechanisms. Accordingly, based on the power functionalism and power conflict theory, this study adopts a dual-path design by considering both the constructive and the destructive nature of power disparity. This method identifies team conflict and team coordination as dual mediators to construct a theoretical research model of the effects of power disparity with the aim of understanding the "double-edged sword effect" of power disparity and testing in further detail whether the two different mechanisms in question are offset or complementary. The reason why team coordination is chosen as the mediator is that power functionalism posits that team coordination is the main path associated with the differential utility of power disparity [10, 16, 17], and the negative effect is also considered to be only caused by internal coordination errors within the team [17]. Specifically, power disparity can help team members understand their positions [10], and clarify their expectations for norms, roles, and expected behaviors based on their positions [15], thereby coordinate the use of task resources and interpersonal interaction [18], and thus promote team innovation. The choice of team conflict as the mediator is based on the power conflict theory, which posits that team conflict is the main process variable interpreting the negative effects of power disparity [2, 11, 17]. Specifically, power disparity leads to opposing interests and views among members of different power hierarchy [19], and intensifies the corresponding power struggle [13], and thereby trigger team conflict, ultimately harming team innovation.

The effects of power disparity on team outcomes also depends on specific boundary conditions [4]. Previous studies on the boundary conditions of power disparity have primarily focused on factors such as team structure [4, 12], task type [20], power-related factors [1, 12] and leadership characteristics [17]. Extant research on power-related factors has mainly emphasized aspects like power distance, the stability of the power structure [20], and power diversity [13], with less attention given to power itself, particularly the key psychological mechanisms underlying the influence of power, such as power cognition. In fact, power cognition, that is, the manner in which members view their own power and that of other members, can significantly influence the effectiveness of power disparity [3]. Power cognition mainly includes the congruence and legitimacy of power. Power legitimacy refers to the team members’ consistent perception of the rationality and legitimacy of the power allocation structure [21], which has a profound effect on the team process [22] and is key to determining whether power disparity operates in the direction of conflict or coordination [2]. Specifically, when power is perceived as legitimate, an imbalanced power allocation is considered to be reasonable. Team members in this situation tend to accept authority and their respective roles without overstepping boundaries [23]. They also tend to engage in more positive interpersonal interactions [21] and provide favorable evaluations of their own potency [22]. These factors help coordinate team members’ behavior and reduce conflicts arising from power disparity. When power is perceived as illegitimate, the imbalanced power allocation is considered to be unreasonable, often leading lower-power members to rebel against the current power allocation and attempting to change it [21]. This will obviously disrupt team members’ expectations, undermine their harmonious relationships, and trigger internal conflicts. Accordingly, power legitimacy may suppress the destructive effect of power disparity on team conflict, enhance the constructive effect of power disparity on team coordination, and ultimately strengthen the positive effect of power disparity on team innovation.

The purpose of this study is twofold. First, based on power functionalism and power conflict theory, we aim to integrate two key paths (coordination and conflict paths) into a single theoretical model to better explain how power disparity affect team innovation, and to understand how these paths coexist and offset each other. Second, from the perspective of key psychological mechanisms of power, we seek to explain how power legitimacy ensures the overall positive effect of power disparity on team innovation. The structure of this paper is as follows: The second section analyzes the mediation and moderation mechanisms of power disparity on team innovation and proposes theoretical hypotheses. The third section introduces the research method, including data sources and measurement. The fourth section presents the empirical results and analysis. The fifth section offers the conclusion and implications.

## 2 Theoretical analysis

### 2.1 The mediating role of team coordination

From the perspective of power functionalism, team coordination is the main process variable associated with power disparity utility [17]. Specifically, power disparity can clarify team members’ roles and positions, support the division of labor within the team, help independent members reach consensus on different opinions, and facilitate interaction and collaboration among team members [9, 13]. It also allows team members to understand their power hierarchy within the team and clarify expectations regarding norms, roles, and behaviors based on their power hierarchy [15, 18, 24]. This provides the team with a well-defined structure and role clarity, enhancing the mutual coordination process. Simultaneously, in cases featuring imbalanced power allocations, the differentiated cognitive mechanisms exhibited by team members complement each other, which is conducive to maintaining cooperation among team members and improve team resource coordination [25]. Additionally, power disparity can create a psychologically safe environment, enhancing members’ willingness to cooperate voluntarily [1], thereby effectively coordinating behavior among team members. Based on their experimental research, Halevy et al. [10] found that imbalanced power allocation improved the coordination within a basketball team.

Coordination refers to the process of integrating group members’ behaviors, knowledge, and goals to attain common objective [26]. It is considered to be an important predictor for outcome variables. In supply chain management, coordination effectively improves the flow of information between supply chain links and enhances decision consistency, thereby reducing decision errors, inefficiencies, and supply chain risks caused by information asymmetry [27]. In advancing the high-quality development of national economies, coordination can promote complementary regional advantages, achieve balanced overall economic growth, and reduce resource waste and social conflicts resulting from regional development imbalances [28]. In autonomous systems, coordination is also crucial as it helps achieve consistent service agreements between systems, improves compatibility among different systems, and avoids strategic conflicts between them [29]. For teams, coordination is also essential. Through coordination, team members gain a deeper understanding of each other’s expertise, allowing them to proactively adjust their behaviors to facilitate the achievement of team goals [30]. Moreover, Coordination helps team members understand the team task objectives, clarify task divisions, and optimize task processes, leading to alignment and synergy in their work. This reduces communication and interaction costs among team members, accelerates the transfer and sharing of knowledge within the team [31], and ultimately promotes team innovation. Based on the comprehensive analysis discussed above, this paper proposes the following hypothesis.

*H*_*1*_: *Team coordination mediates the relationship between power disparity and team innovation performance;specifically*, *power disparity positively affects team innovation performance through team coordination*.

### 2.2 The mediating role of team conflict

Power conflict theory claims that team conflict is an important mediator of the negative effects of power disparity [11, 19]. The effects of power disparity on team conflict have also been confirmed [2, 3, 19]. Specifically, imbalanced power allocation entails an asymmetry in terms of the control of valuable resources within the team [32]; that is, some people have more valuable resources, while others have less. The scarcity and competitiveness of valuable resources inevitably leads to competition, confrontation and conflict [13], power struggles [19], and negative emotions such as perceived unfairness, which worsen interpersonal relationships [12], thereby increasing team conflict. Simultaneously, under conditions of power disparity, low-power individuals tend to seek more power resources, while high-power individuals aim to maintain their existing power advantages [11]. These differences in power motives between the two groups can lead to internal conflict. Furthermore, in studies of multi-team systems, power disparity has been found to increase relationship conflict among stakeholders and reduce their psychological safety [33], with psychological safety being a critical factor in the occurrence of conflict [34]. Greer et al. [35] conducted a field study in a financial company and discovered that in teams with high levels of power disparity, members were more likely to exhibit competitive, aggressive, jealous, and sensitive behaviors, which in turn triggered team conflict.

Team conflict is a common phenomenon in team process that usually affects team innovation negatively. The specific reason for this effect is that team conflict can lead to oppositional interpersonal relationships among members, trigger dissatisfaction, reduce work enthusiasm, distract the team’s attention [36]. It can also generate negative emotions such as depression, dissatisfaction, pressure and tension, and inhibit effective information processing [37], which ultimately harms team innovation. In addition, team conflict leads to mutual exclusion, an unwillingness to cooperate, reluctance to share personal knowledge, all of which are detrimental to team innovation [38]. In summary, power disparity intensifies team conflict, and team conflict negatively affects team innovation. Based on the comprehensive analysis discussed above, this paper proposes the following hypothesis.

*H*_*2*_: *Team conflict mediates the relationship between power disparity and team innovation; specifically*, *power disparity negatively affects team innovation performance through team conflict*.

### 2.3 The moderating role of power legitimacy

Power legitimacy refers to the consistent perception by team members of the rationality and legitimacy of the team power structure, including power levels, disparities, or diversity [2, 3, 21]. It is also an important moderating variable associated with the team power structure [2, 11]. Specifically, when the power structure is perceived as legitimate, team members tend to accept the current power allocation and their own power roles within the team. This acceptance affirms the value of their positions, reduces the likelihood of power struggles, decreases team conflicts, and strengthens team cooperation [10]. Additionally, team members recognize the high cost of disrupting the existing power distribution, leading them to consciously adhere to organizational rules and arrangements, which further promotes internal team coordination [39]. Simultaneously, power legitimacy also helps low-power members identify with the authoritative status of high-power members, leading them to obey the arrangements made by the latter, thus exhibiting less aggression and reducing conflict [40]. Power legitimacy is also regarded as an important signal of the stability of the team power structure. The higher the legitimacy is, the higher the stability of power. In such context, team members recognize the legitimacy of their own power, and do not easily feel questioned and threatened [41]. Even if they are questioned and threatened, their status is not affected, and they engage in more proactive interpersonal interaction behaviors. Even if their power is challenged, it is unlikely to affect their position. This sense of stability encourages members to engage in more proactive interpersonal interactions, which naturally aids in promoting team coordination and reducing conflict. In contrast, when the power structure is perceived as illegitimate, team members tend to question the current power allocation. Lower-power members, in particular, may engage in protest, competition, and other confrontational behaviors to resist or attempt to change the current power allocation [21]. They may also experience more inhibitory psychological states, such as self-doubt, and engage in more passive interpersonal interactions. Power legitimacy is thus conducive to enhancing coordination and alleviating conflict on the team in cases of imbalanced power allocation. Based on the analysis discussed above, this paper proposes the following hypotheses.

*H*_*3*_: *Power legitimacy moderates the relationship between power disparity and team coordination; specifically*, *the stronger the power legitimacy*, *the stronger the positive effect of power disparity on team coordination*.
*H*_*4*_: *Power legitimacy moderates the relationship between power disparity and team conflict; specifically*, *the stronger the power legitimacy*, *the weaker the negative effect of power disparity on team conflict*.

Based on the preceding analysis, this paper posits that the effects of power disparity on team innovation through the dual paths of team coordination and team conflict are moderated by power legitimacy. Specifically, under the influence of power legitimacy, the positive effect of power disparity on team innovation performance through team coordination is strengthened, while the negative effect of power disparity on team innovation performance through team conflict is diminished. Ultimately, this paper proposes two integration hypotheses.

*H*_*5*_: *Power legitimacy moderates the mediating effect of team coordination on the relationship between power disparity and team innovation performance; specifically*, *the stronger power legitimacy*, *the stronger the positive effect of power disparity on team innovation performance through team coordination*.
*H*_*6*_: *Power legitimacy moderates the mediating effect of team conflict on the relationship between power disparity and team innovation performance; specifically*, *the stronger power legitimacy*, *the weaker the negative effect of power disparity on team innovation performance through team conflict*.

**Fig 1:**
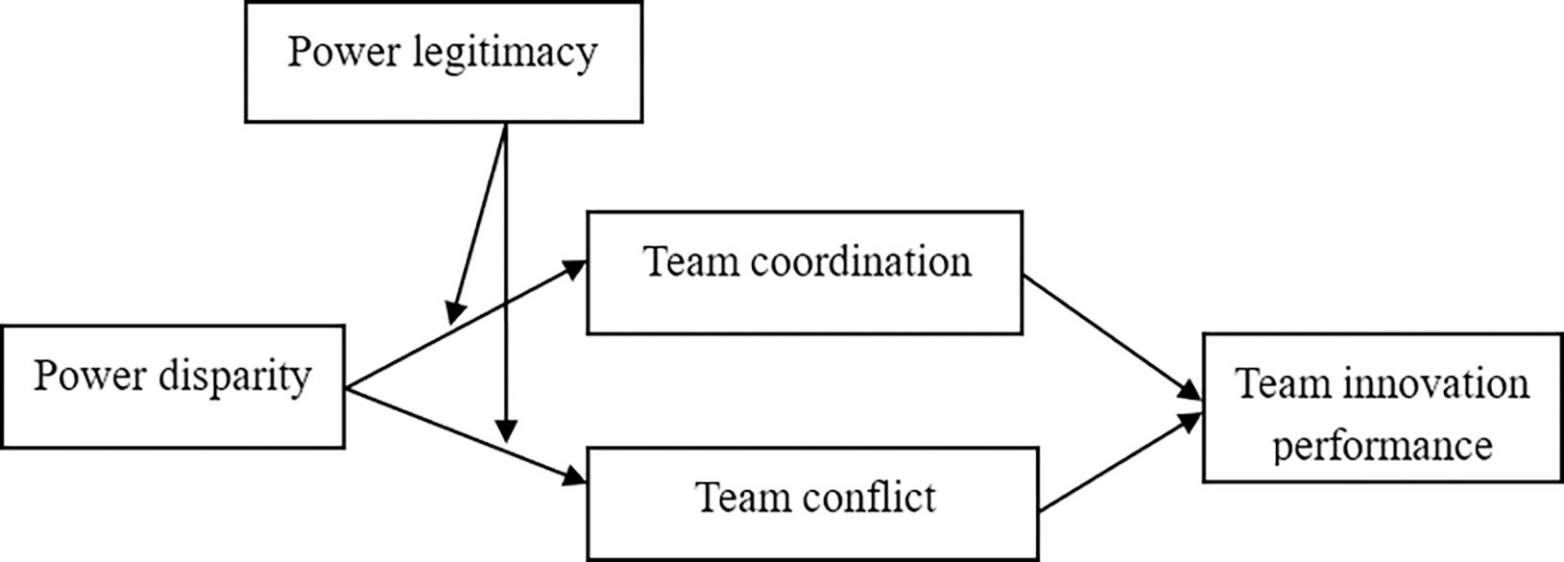
Theoretical model.

Based on this comprehensive analysis, the theoretical model (see Fig 1) of this paper can be obtained.

## 3 Method

### 3.1 Sample and data collection

The research data was collected through a questionnaire survey conducted by the research team between May 2023 and August 2023. The survey covered 93 teams from 27 different types of enterprises in China that were involved in production, marketing, technology research and development, human resources, after-sales service and other departments. Questionnaires were distributed and collected on site. Before distributing the questionnaires, the research team communicated with the participants and addressed any questions they had, ensuring that all participants had a detailed understanding of the concept of power disparity, as well as the research purpose. Additionally, to respect the participants’ rights, questionnaire completion was voluntary, and teams with fewer than three participants were excluded from the study. To reduce the influence of common method bias, a two-stage method was employed to collect data, with an interval of 2 months. During the first stage, 483 questionnaires were distributed to 93 teams, primarily measuring power disparity, team coordination, team conflict, power legitimacy and demographic information. After excluding invalid questionnaires that featured excessively regular answers (This refers to questionnaires that exhibit obvious patterns during the filling process, such as all options being marked the same throughout the entire questionnaire, or highly regular patterns in responses, such as sequences like 11112222) or missing data, 426 valid questionnaires were obtained from 82 teams, for an effective recovery rate of 88.20%. During the second stage, 426 questionnaires were distributed to the 82 teams that had provided valid questionnaires in the first stage to measure team innovation performance. After excluding invalid questionnaires based on the same criteria, 392 valid questionnaires were obtained from 76 teams, for an effective recovery rate of 92.02%. Statistical analysis of the valid questionnaires from the second stage revealed that males accounted for 66.07% and females accounted for 33.93%; participants with college degree or below accounted for 24.49%, participants with an undergraduate degree accounted for 57.14%, and participants with a postgraduate degree accounted for 18.37%; teams with more than 10 people accounted for 6.58%, teams with 5–10 people accounted for 52.63%, and teams with 3–5 people accounted for 40.79%. In terms of tenure, teams with a tenure of more than 3 years accounted for 41.33%, teams with a tenure of 1–3 years accounted for 41.58%, and teams with a tenure of less than 1 year accounted for 17.09%.

### 3.2 Measurements

A 5-point Likert scale was used in all questionnaires.

Power disparity: The rotating questionnaire design method developed by Zhu et al. [2] was used to measure team power disparity. Specifically, each member evaluated the power level of other team members. The evaluation item was as follows: "How much power do I think the colleague has in the team (for example, he or she has asymmetric control over resources and is able to make others implement his or her wishes)". The evaluation score was conducted using a 5-point Likert scale, where 1 indicated "almost not at all" and 5 indicated "very much". Each member’s power level was then calculated by averaging the ratings given by others. After determining each member’s power level, the coefficient of variation was calculated to reflect the degree of team power disparity. A larger coefficient of variation indicates a higher degree of power disparity.

Team coordination: A 5-item scale developed by Fisher et al. [42] was used to measure team coordination, including the item " members of my team effectively adapt their behavior to the actions of other members", "my team effectively coordinates the activities of all members when working to complete the task", "members of my team provide task-related information to other members without being asked", my team proactively helps individual members when they need assistance", "my team monitors the progress of all members’ performance". Cronbach’s α for team coordination was 0.85.

Team conflict: This study employed the classic scale developed by Jehn [43], including relationship conflict and task conflict, with a total of 8 items. The relationship conflict dimension mainly included items such as "how much friction between team members", "how much are personality conflicts evident in your team", "how much tension is there among members in your team", "how much emotional conflict is there among members in your team". The task conflict dimension mainly included 4 items such as "how many conflicts about your work are in the team", "how often do people in your team disagree about opinions regarding the work being done", "how frequently are there conflicts about ideas in your team", "to what extent are there differences of opinion in your team". Cronbach’s α for team conflict was 0.92.

Power legitimacy: A scale developed by Wei and Zhang [44] based on the Chinese context was adopted. This scale mainly included 4 items, such as "I think the power distribution in my team is reasonable", "I think the power distribution in my team is fair", "I think the power distribution in my team is well deserved", "I think the power distribution in my team is convincing". Cronbach’s α for power legitimacy was 0.90.

Team innovation performance: The scale developed by Lovelace et al. [45] was adopted, which mainly included 4 items, such as "our team has a high degree of innovativeness of the team’s product/work", "our team has introduced many innovations or new ideas", "our team has a high degree of overall technical performance", "our team has a high degree of adaptability to change". Cronbach’s α for team innovation performance was 0.87.

Control variables: Given that previous relevant studies have highlighted the fact that team size [46], team diversity [47], and team time [48] may have an effect on the team process and the corresponding results, this study controlled for team size, gender diversity (the Blau index was used to reflect the proportions of men and women on the team) and average team tenure (i.e., the average tenure of team members).

## 4 Research results

### 4.1 Data aggregation

Since this paper focuses on a team-level study, it was necessary to aggregate the individual data to the team level. Data aggregation at the individual level must meet three critical conditions: Rwg>0.70, ICC (1) <0.50 and ICC (2)>0.50. As shown in Table 1, the values for Rwg, ICC (1) and ICC (2) of each variable met the critical conditions, confirming that aggregation was appropriate. This finding shows that the data for these variables were sufficiently varied among teams and exhibited high consistency at the team level.

**Table 1:** The results of data aggregation.

Variables	Rwg	ICC (1)	ICC (2)
Team coordination	0.875	0.214	0.658
Team conflict	0.768	0.256	0.782
Power legitimacy	0.817	0.402	0.675
Team innovation performance	0.806	0.344	0.849

### 4.2 Confirmatory factor analysis

This paper tested five variables, consisting of the power disparity, team coordination, team conflict, power legitimacy and team innovation performance. The results of confirmatory factor analysis are presented in Table 2. As shown in Table 2, the five-factor model exhibited a good fit (*χ*^2^/ *df* = 1.16, RMSEA = 0.04, NNFI = 0. 92, CFI = 0.92, IFI = 0.91). In contrast, the four alternative models showed poor fit and were significantly different from the five-factor model. These results indicate that the study achieved good discriminant validity among the variables.

**Table 2:** The results of confirmatory factor analysis.

Models	*χ*^2^/ *df*	RMSEA	NNFI	CFI	IFI
PD; TCD; TCF; PL; TIP	1.16	0.04	0.92	0.92	0.91
PD; TCD; TCF; PL+TIP	1.45	0.18	0.83	0.83	0.83
PD; TCD; TCF+PL+TIP	1.95	0.25	0.79	0.78	0.78
PD; TCD+TCF+PL+TIP	2.26	0.34	0.72	0.71	0.72
PD+TCD+TC+PL+TIP	3.07	0.38	0.63	0.63	0.64

Note: PD = power disparity; TCD = team coordination; TCF = team conflict; PL = power legitimacy; TIP = team innovation performance.

### 4.3 Descriptive statistics

Descriptive statistical analysis of eight variables, including control variables, was conducted using SPSS 23.0. The analysis provided the mean values, standard deviations, and correlation coefficients for these variables (see Table 3). According to Table 3, power disparity is positively correlated with both team coordination (*r* = 0.442, *P*<0.01) and team conflict (*r* = 0.219, *P*<0.01). team coordination is positively correlated with team innovation performance (*r* = 0.466, *P*<0.01), while team conflict is negatively correlated with team innovation performance (*r* = -0.401, *P*<0.01). Additionally, team coordination is not significantly related to team conflict (*r* = 0.027), indicating that there was no problem with the selection of mediators.

**Table 3:** The results of descriptive statistics.

Variables	M	SD	TS	GD	TT	PD	TCD	TCF	PL	TP
TS	5.158	1.200	1.000							
GD	0.240	0.073	-0.086	1.000						
TT	1.618	0.341	-0.048	-0.117	1.000					
PD	0.216	0.063	0.123	0.055	0.005	1.000				
TCD	3.976	0.536	-0.138	-0.145	0.008	0.442**	1.000			
TCF	2.850	0.562	0.021	-0.051	-0.112	0.219**	0.027	1.000		
PL	4.108	0.566	-0.131	0.014	0.009	0.236**	0.398**	-0.189**	1.000	
TIP	3.845	0.569	-0.028	0.015	-0.088	0.171**	0.466**	-0.401**	0.311**	1.000

Note: *P<0.05

**P<0.01; TS = team size; GD = gender diversity; TT = team tenure; PD = power disparity; TCD = team coordination; TCF = team conflict; PL = power legitimacy; TIP = team innovation performance.

### 4.4 Hypothesis testing

#### 4.4.1 The mediating effect of team coordination

First, a linear regression of team innovation performance on power disparity was conducted to obtain M_1_. The results indicated that the total effect of power disparity on team innovation performance is significantly positive (*β* = 0.128, *P*<0.05). Second, a linear regression of team coordination on power disparity was conducted to obtain M_4_. The results of M_4_ indicate that power disparity has a significantly positive effect on team coordination (*β* = 0.352, *P*<0.01). Furthermore, team innovation performance was regressed on both power disparity and team coordination simultaneously to obtain M_2_. The results of M_2_ revealed that team coordination has a significantly positive effect on team innovation performance (*β* = 0.507, *P*<0.01), while the effect of power disparity on team innovation performance remains significant (*β* = 0.127, *P*<0.05). These findings indicate that team coordination partially mediates the relationship between power disparity and team innovation performance, thereby supporting H_1_.

#### 4.4.2 The mediating effect of team conflict

To analyze the mediating effect of team conflict, a linear regression of team conflict on power disparity was conducted to obtain M_7_. The results of M_7_ show that power disparity has a significantly positive effect on team conflict (*β* = 0.210, *P*<0.01). Subsequently, team innovation performance was regressed on both power disparity and team conflict simultaneously to obtain M_3_. The results of M_3_ indicate that team conflict significantly affects team innovation performance (*β* = -0.282, *P*<0.01), and the effect of power disparity on team innovation performance remains significant (*β* = 0.128, *P*<0.05). These findings suggest that team conflict partially mediates the relationship between power disparity and team innovation performance, thereby supporting H_2_.

To further verify the significance of the mediating effects through different paths,

a Bootstrap analysis with 2,000 samples was conducted. As shown in Table 4, the mediating effect size for team coordination is 0.044, and for team conflict, it is -0.022. Both mediating effects are significant, with their 95% CI not including 0. Therefore, hypotheses H_1_ and H_2_ are further verified. Notably, the positive mediating effect of team coordination is greater than the negative mediating effect of team conflict, indicating that the positive path of team coordination can offset the negative path of team conflict.

**Table 4:** The comparative analysis of mediating effects.

	Pathways	Effect size	SE	95% CI
Total effect	All	0.128	0.045	[0.012, 0.355]
Direct effect	PD—TIP	0.106	0.033	[0.019, 0.139]
Indirect effect	PD—TCD—TIP	0.044	0.019	[0.008, 0.031]
	PD—TCF—TIP	-0.022	0.017	[-0.027, -0.010]

Note: PD = power disparity; TCD = team coordination; TCF = team conflict; TIP = team innovation performance.

#### 4.4.3 The moderating effect of power legitimacy

**Fig 2:**
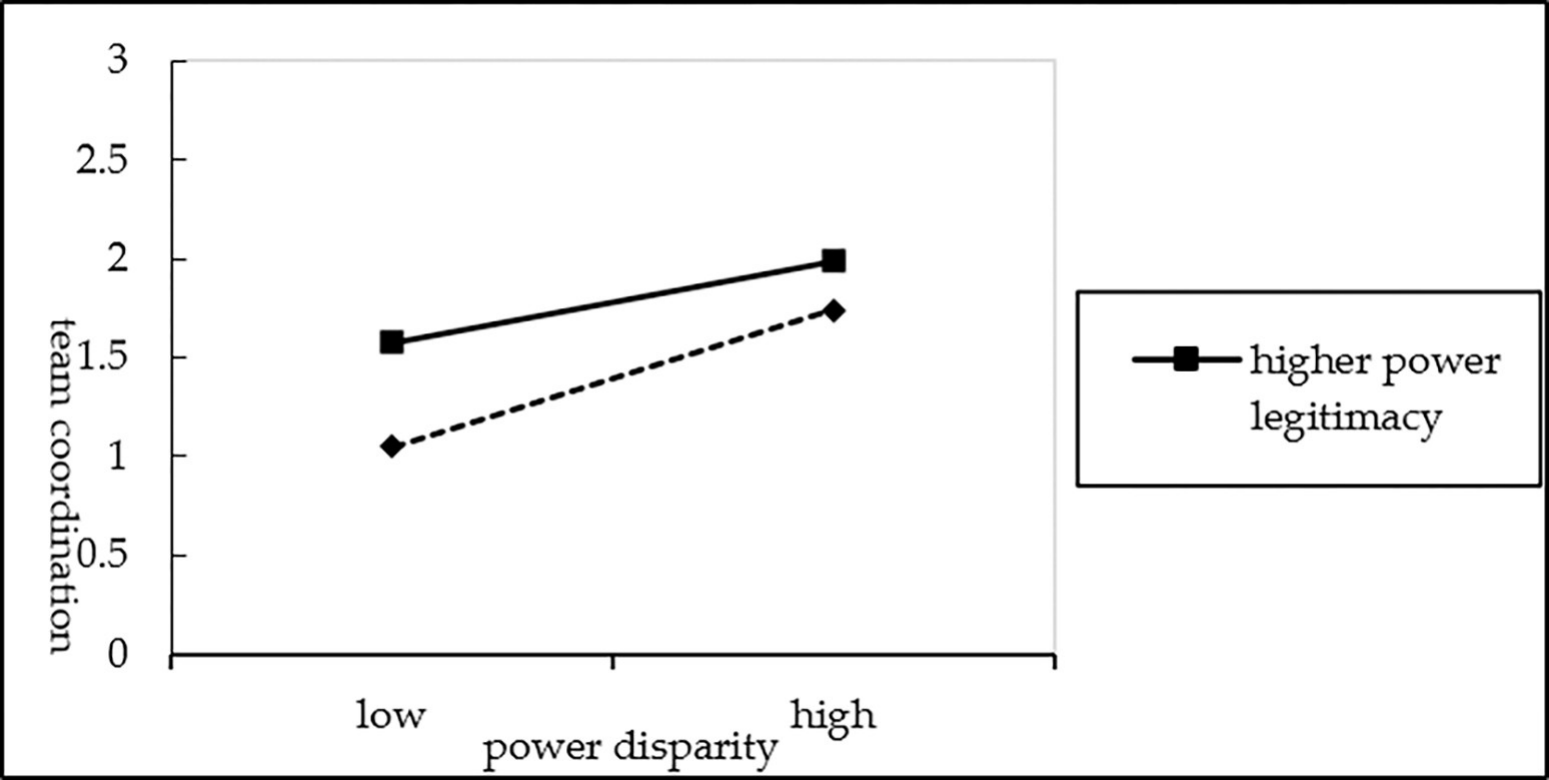
The moderating effects of power legitimacy on power disparity and team coordination.

The analysis consisted of two parts. First, the moderating effects of power legitimacy on the relationship between power disparity and team coordination was computed. On the basis of M_4_, power legitimacy was added to the regression to create M_5_. Then, an interaction term (power disparity × power legitimacy) was introduced in M_6_. The results of M_6_ show that the interaction item has a positive effect on team coordination (*β =* 0.212, *P<0*.*01*), indicating that power legitimacy positivly moderate the relationship between power disparity and team coordination, thereby supporting H_3_ (see Fig 2).

**Fig 3:**
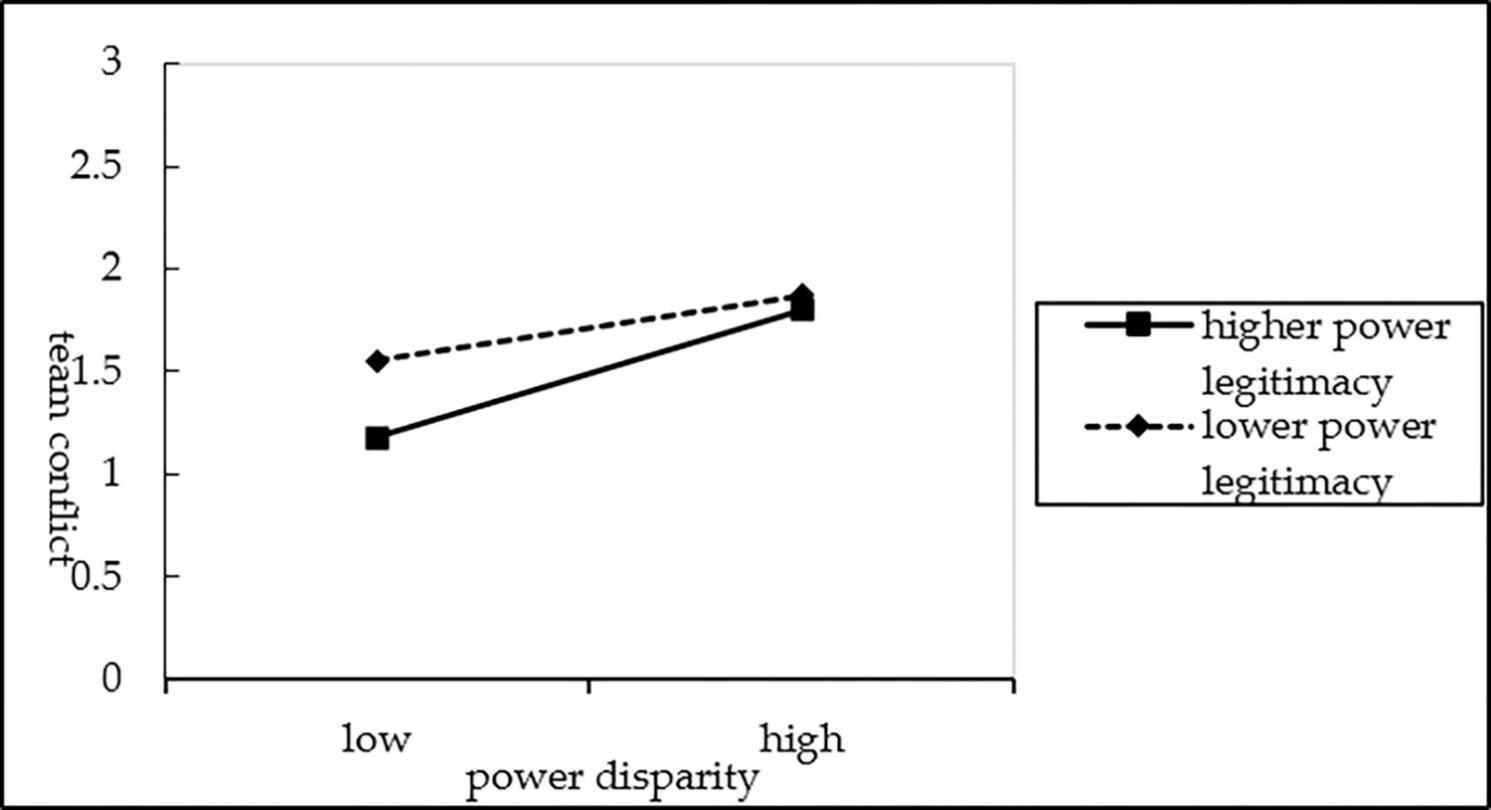
The moderating effects of power legitimacy on power disparity and team conflict.

Second, the moderating effects of power legitimacy on the relationship between power disparity and team conflict was analyzed. On the basis of M_7_, power legitimacy was added to the regression to obtain M_8_. Then, an interaction term (power disparity × power legitimacy) was introduced in M_9_. The results of M_9_ show that the interaction item has a negative effect on team conflict (*β = -*0.185, *P<0*.*01*), indicating that power legitimacy has a negative moderating effect on the relationship between power disparity and team conflict, thereby supporting H_4_ (see Fig 3). Table 5 presents the results of the computations for M_1_ to M_9_.

**Table 5:** The results of mediating effect and moderating effect analysis.

Variables	TIP			TCD			TCF		
	M_1_	M_2_	M_3_	M_4_	M_5_	M_6_	M_7_	M_8_	M_9_
TZ	-0.037	0.040	-0.046	-0.053	-0.059	-0.060	0.011	0.023	0.023
GD	0.120	0.202	0.092	-0.061	-0.154	-0.149	-0.062	-0.062	-0.066
TT	-0.076	-0.067	-0.070	-0.018	-0.016	-0.014	-0.119	-0.119	-0.121
PD	0.128*	0.127*	0.128*	0.352**	0.324**	0.164**	0.210**	0.273**	0.177**
TCD		0.507**							
TCF			-0.282**						
PL					0.387**	0.472**		0.249*	0.225**
PD*PL						0.212**			-0.185**
R^2^	0.044	0.290	0.034	0.043	0.185	0.186	0.017	0.019	0.020
△R^2^	-0.010	0.239	-0.024	-0.010	0.127	0.115	-0.038	-0.051	-0.066

Note: *P<0.05

**P<0.01; TS = team size; GD = gender diversity; TT = team tenure; PD = power disparity; TCD = team coordination; TCF = team conflict; PL = power legitimacy; TIP = team innovation performance.

#### 4.4.4 Moderated mediation test

To test H_5_, which examines the moderating effect of team coordination mediation, a bootstrap analysis with 2,000 samples was conducted. The results show that the 95% CI is [0.011, 0.027] (see Table 6), indicating that the moderated mediation effect is significant. Additionally, the Monte Carlo method was used for further validation. The results showed that the 95% CI of the moderating effect of power legitimacy on the mediator of team coordination is [0.005, 0.019], confirming that the moderating effect related to the mediation of team coordination is significant, thereby supporting H_5_.

**Table 6:** The results of the moderating effect of team coordination mediation.

	EV	SE	95% CI
higher power legitimacy	0.027	0.018	[0.020, 0.041]
lower power legitimacy	0.021	0.023	[0.008, 0.028]
the moderated mediator	0.023	0.029	[0.011, 0.027]

Similarly, to test H_6_, which examines the moderating effect of team conflict mediation, a bootstrap analysis with 2,000 samples was conducted. The results show that the 95% CI is [0.007, 0.046] (see Table 7), indicating that the moderated mediation effect is significant. Additionally, The Monte Carlo method was employed for further validation. The results revealed that the 95% CI of the moderating effect of power legitimacy on the mediator of team conflict is [0.013, 0.058], confirming that the moderating effect associated with the mediating effect of team conflict is significant, thereby supporting H_6_.

**Table 7:** The results of the moderating effect of team conflict mediation.

	EV	SE	95% CI
higher power legitimacy	0.056	0.039	[0.012, 0.055]
lower power legitimacy	0.060	0.014	[0.014, 0.072]
the moderated mediator	0.033	0.054	[0.007, 0.046]

## 5 Discussion and implications

### 5.1 Discussion

First, with regard to innovation, both centralized and decentralized power allocation have dual effects, that is, power disparity has a "double-edged sword" effect. From the perspective of power functionalism, power disparity can facilitate labor division, unify divergent opinions, enhance team members interactions, and ultimately boost team innovation performance. Conversely, from the perspective of power conflict theory, power disparity can incite power struggle, exacerbate team conflict, strain interpersonal relationships, and thereby harm team innovation performance. These findings align with previous views on the effects of power disparity, though the differing perspectives stem from distinct theoretical frameworks.

Second, the positive effect of team coordination path offsets the negative effect of team conflict path, resulting in an overall positive effect of power disparity on team innovation performance. This finding contradicts the meta-analysis view that the power conflict theory overwhelmingly outweighs power functionalism [17]. The discrepancy may be attributed to the nature of the innovative tasks undertaken by the team, as contingency theory suggests that the effect of power disparity is moderated by task type [49]. For simple tasks, innovation does not require extensive input from team members, allowing power disparity to function effectively [4]. This aligns with the reality that most Chinese companies invest less in R&D, with high-quality invention patents accounting for only a small portion of the total number of authorized patents.

Third, this study confirmed the moderating effect of power legitimacy. When power legitimacy is perceived, team members view the current power allocation as reasonable and legitimate, leading them to accept their roles, adhere to organizational arrangements, and refrain from challenging authority. This reduces team conflict and enhances mutual cooperation, thereby strengthening the positive effect of power disparity on team coordination and diminishing its negative effect on team conflict. Moreover, power legitimacy moderates the mediating effects of team coordination and team conflict on the relationship between power disparity and team innovation performance. Specifically, the higher the level of power legitimacy, the stronger the positive effect of power disparity on team innovation performance through team coordination, and the weaker the negative effect through team conflict.

### 5.2 Theoretical contributions

First, this study constructed a dual-path model of power disparity and systematically explained the uncertain effects of power disparity on team innovation performance. Previous meta-analyses have indicated that power disparity has a "double-edged sword" effect on team innovation performanc [17]. However, research has largely focused on the main effects of power disparity, often overlooking the distinct paths through which these effects operate. Few studies have investigated both the constructive and destructive paths of power disparity within the same theoretical model, which has led to an overemphasis on one of these mechanisms [12]. In response, some scholars have proposed developing a theoretical model of power disparity that incorporates multiple paths [50]. This study, therefore, explored both the constructive effect of power disparity on team innovation performance through team coordination and the destructive effect through team conflict, providing a comprehensive explanation of the uncertain relationship between these paths.

Second, this study expanded the research on the boundary conditions of power disparity. Previous studies have seldom focused on power-related psychological factors, despite their potential to influence individuals’ willingness to redistribute power, significantly affect the effectiveness of power structure [3], and determine whether power disparity leads to conflict or coordination [2], ultimately affecting team innovation performance. In response, this study explored the moderating effect of power legitimacy on the utility of power disparity. Empirical research revealed that power legitimacy enhances the positive effect of power disparity on team innovation performance through team coordination while mitigating its negative effect through team conflict. These findings underscore the importance of power cognition as a power psychological factor in understanding power disparity, enriching the existing research on power disparity, and offering new perspectives for future research studies in this area.

### 5.3 Practical implications

First, power allocation should be approached dialectically. This study confirmed that power disparity can both positively affect team innovation performance through team coordination and negatively affect team innovation performance through team conflict. Therefore, organizations should strategically allocate power by leveraging its constructive aspects. This involves consciously distributing power resources in a differentiated manner, clarifying superior-subordinate relationships, establishing a clear power hierarchy, and empowering leaders to optimize internal relationship chains. This approach helps foster cooperation and reduce internal conflicts. At the same time, it is important to address the negative effects of power disparity. Organizations should mitigate power blockades, offer promotion opportunities to lower-power members, and manage power struggles that may arise from hierarchical structures.

Second, the importance of power legitimacy in designing of the power structure should be emphasized. Power legitimacy, which reflects team members’ perception of the fairness of the power allocation, significantly affects the effectiveness of power allocation [11] and acts as an important moderator of the power structure [2, 3]. this study confirmed that when power legitimacy is perceived, team members recognize the rationality of the current power structure and accept their own roles, which reduces internal conflict, enhances cooperation, and ultimately improves team innovation performance. Therefore, enterprises should take effective measures to enhance team members’ perception of power legitimacy. First, They should ensure the fairness, transparency and rationality in resource allocation to boost recognition and acceptance of the power structure. Second, they should value each team member, provide positive feedback to contributors, and avoid situations where individuals with high power take undue credit.

### 5.4 Limitations and directions for future research

Although this study yields valuable insights, it has some limitations. First, It only examined the effect of power disparity on team innovation performance from a dual-path perspective at the team level. however, some studies have reported that this effect results from the simultaneous interaction between power disparity and process variables at both individual and team levels. Therefore, future research should develop a comprehensive theoretical model that incorporates multilevel principles. Second, while this study identified team conflict as a key process variable, it considered team conflict only as a single-dimensional variable. In fact, team conflict comprises various types, each affecting team outcomes differently [2]. Future research should investigate the differential utility of power disparity in relation to various types of conflict to understand how these differences affect team outcomes. Third, this study conceptualizes power disparity primarily from a resource-based perspective, focusing on formal power derived from positions and individual roles. However, within the context of Chinese Confucian culture, power is not only derived from positions and individual roles but is also closely related to interpersonal relationships. Some scholars have defined relational power based on Chinese cultural contexts [51]. As an informal form of power, relational power can influence others through personal connections and interactions, affecting resource allocation, decision-making, and the functioning of social structures. Therefore, future research should expand the concept of power disparity to include sources of power relevant to the Chinese cultural context, particularly studies on relational power.

## 6 Conclusion

This study confirmed the double-edged sword effect of power disparity; that is, according to the same theoretical model, power disparity not only positively affects team innovation performance through team coordination but also negatively affects team innovation performance through team conflict. However, under the influence of power legitimacy, the negative effect of power disparity is suppressed, and the positive effect is strengthened, thus ensuring that power disparity has a positive effect on team innovation performance. The findings provide valuable insights for optimizing power allocation in the team innovation process.

## Supporting information

S1 File The reliability of variables. (DOCX)

S2 File The descriptive statistics of variables. (DOCX)

S3 File The regression of Model 1 (TIP—PD). (DOCX)

S4 File The regression of Model 2 (TIP—PD+TCD). (DOCX)

S5 File The regression of Model 3 (TIP—PD+TCF). (DOCX)

S6 File The regression of Model 4 (TCD—PD). (DOCX)

S7 File The regression of Model 5 (TCD—PD+PL). (DOCX)

S8 File The regression of Model 6 (TCD—PD+PL+PD*PL). (DOCX)

S9 File The regression of Model 7 (TCF—PD). (DOCX)

S10 File The regression of Model 8 (TCF—PD+PL). (DOCX)

S11 File The regression of Model 9 (TCF—PD+PL+PD*PL). (DOCX)

S12 File The questionnaire. (DOCX)
