# Supplementary material for: Centralization or decentralization? Power allocation in team innovation management
Source: PLoS One. 2024 Oct 28;19(10):e0310719. doi: 10.1371/journal.pone.0310719 (PMC11516181; doi:10.1371/journal.pone.0310719)
Supplement: S1 File — (DOCX) [file pone.0310719.s001.docx]

The reliability of variables

1. TCD (team coordination)

| **Case Processing Summary** | | | |
| --- | --- | --- | --- |
|  | | N | % |
| Case | Valid | 76 | 100.0 |
|  | Excluded^a^ | 0 | .0 |
|  | Total | 76 | 100.0 |
| a. Listwise deletion based on all variables in the procedure. | | | |

| **Reliability Statistics** | | |
| --- | --- | --- |
| CCronbach's Alpha | \Cronbachs Alpha Based onstandardized items | 项N of items |
| ..862 | ..853 | 55 |

| **Item Statistics** | | | |
| --- | --- | --- | --- |
|  | Mean | Std.Deviation | N |
| TCD1 | 3.5132 | .90175 | 76 |
| TCD2 | 4.4211 | .67849 | 76 |
| TCD3 | 3.9868 | 1.05190 | 76 |
| TCD4 | 3.7237 | .96054 | 76 |
| TCD5 | 4.2368 | .72789 | 76 |

2. TCF (team conflict)

| **Reliability Statistics** | | |
| --- | --- | --- |
| CCronbach's Alpha | \Cronbachs Alpha Based onstandardized items | 项N of items |
| ..940 | ..921 | 5 8 |

| **Item Statistics** | | | |
| --- | --- | --- | --- |
|  | Mean | Std.Deviation | N |
| TCF1 | 2.5132 | .85625 | 76 |
| TCF2 | 2.9737 | .86369 | 76 |
| TCF3 | 2.5789 | .92755 | 76 |
| TCF4 | 3.0526 | .93659 | 76 |
| TCF5  TCF6  TCF7  TCF8 | 2.9211  2.8684  2.9737  2.9211 | .96282  .77187  .79956  .93471 | 76  76  76  76 |

3. PL (power legitimacy)

| **Reliability Statistics** | | |
| --- | --- | --- |
| CCronbach's Alpha | \Cronbachs Alpha Based onstandardized items | 项N of items |
| ..919 | ..902 | 54 |

| **Item Statistics** | | | |
| --- | --- | --- | --- |
|  | Mean | Std.Deviation | N |
| PL1 | 3.6184 | 1.07042 | 76 |
| PL2 | 4.3158 | .73413 | 76 |
| PL3 | 4.2763 | .79328 | 76 |
| PL4 | 4.1711 | .73735 | 76 |

4. TIP (team innovation performance)

| **Reliability Statistics** | | |
| --- | --- | --- |
| CCronbach's Alpha | \Cronbachs Alpha Based onstandardized items | 项N of items |
| ..886 | ..873 | 54 |

| **Item Statistics** | | | |
| --- | --- | --- | --- |
|  | Mean | Std.Deviation | N |
| TIP1 | 4.1184 | .89394 | 76 |
| TIP2 | 4.2237 | .85788 | 76 |
| TIP3 | 4.3553 | .76077 | 76 |
| TIP4 | 4.5132 | .73925 | 76 |
