# Supplementary material for: Centralization or decentralization? Power allocation in team innovation management
Source: PLoS One. 2024 Oct 28;19(10):e0310719. doi: 10.1371/journal.pone.0310719 (PMC11516181; doi:10.1371/journal.pone.0310719)
Supplement: S2 File — (DOCX) [file pone.0310719.s002.docx]

The descriptive statistics of variables

1. The Mean, Standard Deviationand and Variance of variables

| **Descriptive Statistics** | | | | | | |
| --- | --- | --- | --- | --- | --- | --- |
|  | N | Minimun | Maximun | Mean | Std. Deviation | Var |
| PD | 76 | .10 | .36 | .2161 | .06381 | .004 |
| GD | 76 | .11 | .41 | .2403 | .07257 | .005 |
| TT | 76 | .39 | 2.76 | 1.6182 | .34104 | .116 |
| TS | 76 | 3.00 | 13.00 | 5.1581 | 1.20060 | 1.441 |
| TCD | 76 | 1.80 | 5.00 | 3.9763 | .53563 | .287 |
| PL | 76 | 2.50 | 5.00 | 4.1086 | .56618 | .321 |
| TCF | 76 | 1.75 | 4.63 | 2.8503 | .56237 | .316 |
| TIP | 76 | 2.25 | 5.00 | 3.8446 | .56932 | .324 |
| Valid N (listwise) | 76 |  |  |  |  |  |

2. The correlation analysis

| **correlations** | | | | | | | | | | |
| --- | --- | --- | --- | --- | --- | --- | --- | --- | --- | --- |
|  | | TS | GD | TT | PD | TCD | TCF | PL | TIP |  |
| TS | Pearson correlation | 1 | -.086 | -.048 | .123 | -.138 | .021 | -.231^*^ | -.028 |  |
|  | Sig.（2-tailed） |  | .463 | .683 | .289 | .235 | .859 | .045 | .427 |  |
|  | N | 76 | 76 | 76 | 76 | 76 | 76 | 76 | 76 |  |
| GD | Pearson correlation | -.086 | 1 | -.117 | .055 | -.145 | -.051 | .014 | .015 |  |
|  | Sig.（2-tailed） | .463 |  | .313 | .180 | .211 | .662 | .905 | .606 |  |
|  | N | 76 | 76 | 76 | 76 | 76 | 76 | 76 | 76 |  |
| TT | Pearson correlation | -.048 | -.117 | 1 | .005 | .008 | -.112 | .009 | -.088 |  |
|  | Sig.（2-tailed） | .683 | .313 |  | .964 | .944 | .335 | .936 | .545 |  |
|  | N | 76 | 76 | 76 | 76 | 76 | 76 | 76 | 76 |  |
| PD | Pearson correlation | .123 | .055 | .005 | 1 | .442^**^ | -.219^**^ | .236^**^ | .171^**^ |  |
|  | Sig.（2-tailed） | .289 | .180 | .964 |  | .000 | .001 | .001 | .001 |  |
|  | N | 76 | 76 | 76 | 76 | 76 | 76 | 76 | 76 |  |
| TCD | Pearson correlation | -.138 | -.145 | .008 | .442^**^ | 1 | .027 | .398^**^ | .466^**^ |  |
|  | Sig.（2-tailed） | .235 | .211 | .944 | .000 |  | .818 | .000 | .001 |  |
|  | N | 76 | 76 | 76 | 76 | 76 | 76 | 76 | 76 |  |
| TCF | Pearson correlation | .021 | -.051 | -.112 | -.219^**^ | .027 | 1 | .189^**^ | -.401^**^ |  |
|  | Sig.（2-tailed） | .859 | .662 | .335 | .001 | .818 |  | .000 | .000 |  |
|  | N | 76 | 76 | 76 | 76 | 76 | 76 | 76 | 76 |  |
| PL | Pearson correlation | -.131 | .014 | .009 | .236^**^ | .398^**^ | .189^**^ | 1 | .311^**^ |  |
|  | Sig.（2-tailed） | .055 | .905 | .936 | .001 | .000 | .000 |  | .000 |  |
|  | N | 76 | 76 | 76 | 76 | 76 | 76 | 76 | 76 |  |
| TIP | Pearson correlation | -.028 | .015 | -.088 | .171^**^ | .466^**^ | -.401^**^ | .311^**^ | 1 |  |
|  | Sig.（2-tailed） | .427 | .606 | .545 | .001 | .001 | .000 | .000 |  |  |
|  | N | 76 | 76 | 76 | 76 | 76 | 76 | 76 | 76 |  |
| *. Correlation is significant at the 0.05 level (2-tailed). | | | | | | | | | | |
| **. Correlation is significant at the 0.01 level (2-tailed). | | | | | | | | | | |
