# Supplementary material for: Centralization or decentralization? Power allocation in team innovation management
Source: PLoS One. 2024 Oct 28;19(10):e0310719. doi: 10.1371/journal.pone.0310719 (PMC11516181; doi:10.1371/journal.pone.0310719)
Supplement: S3 File — (DOCX) [file pone.0310719.s003.docx]

The regression of Model 1 (TIP—PD)

| **Entered／Removed variables^a^** | | | |
| --- | --- | --- | --- |
| Model | Entered variables | Removed variables | Method |
| 1 | PD, TT, TS, GD^b^ | . | Enter |
| a. Dependent Variable: TIP | | | |
| b. All requested variables have been entered. | | | |

| **Model Summary^b^** | | | | | | | | | | | |
| --- | --- | --- | --- | --- | --- | --- | --- | --- | --- | --- | --- |
| Model | R | R Square | Adjusted R Square | Std Error of the Estimate | Change Statistics | | | | | Durbin-Watson |  |
|  |  |  |  |  | R Square  Change | F Change | df1 | df2 | Sig. F Change |  |  |
| 1 | .210^a^ | .044 | -.010 | .62344 | .044 | 7.527 | 4 | 71 | .007 | 1.757 |  |
| a. Predictive Variables: (Constant), PD, TT, TS, GD. | | | | | | | | | | | |
| b. Dependent Variable: TIP | | | | | | | | | | | |

| **Anova^a^** | | | | | | | | | | | | |  |  |  |
| --- | --- | --- | --- | --- | --- | --- | --- | --- | --- | --- | --- | --- | --- | --- | --- |
| Model | | Sum of Squares | | | df | | Mean Square | | F | | Sig. | |  |  |  |
| 1 | Regression | .819 | | | 4 | | .205 | | 7.527 | | .007^b^ | |  |  |  |
|  | Residual | 27.596 | | | 71 | | .389 | |  | |  | |  |  |  |
|  | Total | 28.414 | | | 75 | |  | |  | |  | |  |  |  |
| a. Dependent Variable: TIP | | | | | | | | | | | | |  |  |  |
| b. Predictive Variables: (Constant), PD, TT, TS, GD. | | | | | | | | | | | | |  |  |  |
| **Coefficients^a^** | | | | | | | | | | | | |  |  |  |
| Model | | | | Unstandardized Coefficients | | | standardized Coefficients | | t | | Sig. | | 95.0% CI For B | | |
|  |  |  |  | B | Std. Error | | Beta | |  |  |  |  | Lower Bound | | Upper Bound |
| 1 | | (Constant) | | 4.336 | .457 | |  | | 9.491 | | .000 | | 3.425 | | 5.247 |
|  |  | TS | | -.030 | .033 | | -.037 | | -.910 | | .366 | | -.096 | | .036 |
|  |  | GD | | .206 | 1.018 | | .120 | | .203 | | .840 | | -1.823 | | 2.236 |
|  |  | TT | | -.083 | .134 | | -.076 | | -.622 | | .536 | | -.351 | | .184 |
|  |  | PD | | 1.113 | 1.154 | | .128 | | .965 | | .018 | | 1.187 | | 3.414 |
| a. Dependent Variable: TIP | | | | | | | | | | | | | | | |
