# Supplementary material for: Centralization or decentralization? Power allocation in team innovation management
Source: PLoS One. 2024 Oct 28;19(10):e0310719. doi: 10.1371/journal.pone.0310719 (PMC11516181; doi:10.1371/journal.pone.0310719)
Supplement: S4 File — (DOCX) [file pone.0310719.s004.docx]

The regression of Model 2 (TIP—PD+TCD)

| **Entered／Removed variables^a^** | | | |
| --- | --- | --- | --- |
| Model | Entered variables | Removed variables | Method |
| 1 | TCD,TT,  PD, TS, GD^b^ | . | Enter |
| a. Dependent Variable: TIP | | | |
| b. All requested variables have been entered. | | | |

| **Model Summary^b^** | | | | | | | | | | | |
| --- | --- | --- | --- | --- | --- | --- | --- | --- | --- | --- | --- |
| Model | R | R Square | Adjusted R Square | Std Error of the Estimate | Change Statistics | | | | | Durbin-Watson |  |
|  |  |  |  |  | R Square  Change | F Change | df1 | df2 | Sig. F Change |  |  |
| 1 | .538^a^ | .290 | .239 | .58029 | .290 | 2.876 | 5 | 70 | .020 | 1.963 |  |
| a. Predictive Variables: (Constant),TCD, TT, PD, TS, GD. | | | | | | | | | | | |
| b. Dependent Variable: TIP | | | | | | | | | | | |

| **Anova^a^** | | | | | | | | | | | | |  |  |  |
| --- | --- | --- | --- | --- | --- | --- | --- | --- | --- | --- | --- | --- | --- | --- | --- |
| Model | | Sum of Squares | | | df | | Mean Square | | F | | Sig. | |  |  |  |
| 1 | Regression | 4.843 | | | 5 | | .969 | | 2.876 | | .020^b^ | |  |  |  |
|  | Residual | 23.572 | | | 70 | | .337 | |  | |  | |  |  |  |
|  | Total | 28.414 | | | 75 | |  | |  | |  | |  |  |  |
| a. Dependent Variable: TIP | | | | | | | | | | | | |  |  |  |
| b. Predictive Variables: (Constant), TCD, TT, PD, TS, GD. | | | | | | | | | | | | |  |  |  |
| **Coefficients^a^** | | | | | | | | | | | | |  |  |  |
| Model | | | | Unstandardized Coefficients | | | standardized Coefficients | | t | | Sig. | | 95.0% CI For B | | |
|  |  |  |  | B | Std. Error | | Beta | |  |  |  |  | Lower Bound | | Upper Bound |
| 1 | | (Constant) | | 2.337 | .718 | |  | | 3.257 | | .002 | | .906 | | 3.769 |
|  |  | TS | | .014 | .031 | | .040 | | -.440 | | .661 | | -.076 | | .049 |
|  |  | GD | | .731 | .960 | | .086 | | .762 | | .449 | | -1.183 | | 2.645 |
|  |  | TT | | -.076 | .125 | | -.066 | | -.605 | | .547 | | -.325 | | .174 |
|  |  | PD  TCD | | 1.105  .742 | 1.074  .128 | | .127  .507 | | 1.029  3.457 | | .027  .001 | | 2.036  .187 | | 3.247  .698 |
| a. Dependent Variable: TIP | | | | | | | | | | | | | | | |
