# Supplementary material for: Centralization or decentralization? Power allocation in team innovation management
Source: PLoS One. 2024 Oct 28;19(10):e0310719. doi: 10.1371/journal.pone.0310719 (PMC11516181; doi:10.1371/journal.pone.0310719)
Supplement: S5 File — (DOCX) [file pone.0310719.s005.docx]

The regression of Model 3 (TIP—PD+TCF)

| **Entered／Removed variables^a^** | | | |
| --- | --- | --- | --- |
| Model | Entered variables | Removed variables | Method |
| 1 | TCF,PD, TT, TS, GD^b^ | . | Enter |
| a. Dependent Variable: TIP | | | |
| b. All requested variables have been entered. | | | |

| **Model Summary^b^** | | | | | | | | | | | |
| --- | --- | --- | --- | --- | --- | --- | --- | --- | --- | --- | --- |
| Model | R | R Square | Adjusted R Square | Std Error of the Estimate | Change Statistics | | | | | Durbin-Watson |  |
|  |  |  |  |  | R Square  Change | F Change | df1 | df2 | Sig. F Change |  |  |
| 1 | .184^a^ | .034 | -.024 | .62726 | .034 | 6.444 | 5 | 70 | .016 | 1.757 |  |
| a. Predictive Variables: (Constant), TCF, PD, TT, TS, GD. | | | | | | | | | | | |
| b. Dependent Variable: TIP | | | | | | | | | | | |

| **Anova^a^** | | | | | | | | | | | | |  |  |  |
| --- | --- | --- | --- | --- | --- | --- | --- | --- | --- | --- | --- | --- | --- | --- | --- |
| Model | | Sum of Squares | | | df | | Mean Square | | F | | Sig. | |  |  |  |
| 1 | Regression | .873 | | | 5 | | .175 | | 6.444 | | .016^b^ | |  |  |  |
|  | Residual | 27.542 | | | 70 | | .393 | |  | |  | |  |  |  |
|  | Total | 28.414 | | | 75 | |  | |  | |  | |  |  |  |
| a. Dependent Variable: TIP | | | | | | | | | | | | |  |  |  |
| b. Predictive Variables: (Constant), TCF, PD, TT, TS, GD. | | | | | | | | | | | | |  |  |  |
| **Coefficients^a^** | | | | | | | | | | | | |  |  |  |
| Model | | | | Unstandardized Coefficients | | | standardized Coefficients | | t | | Sig. | | 95.0% CI For B | | |
|  |  |  |  | B | Std. Error | | Beta | |  |  |  |  | Lower Bound | | Upper Bound |
| 1 | | (Constant) | | 4.183 | .617 | |  | | 6.781 | | .000 | | 2.953 | | 5.413 |
|  |  | TS | | -.030 | .033 | | -.046 | | -.909 | | .367 | | -.097 | | .036 |
|  |  | GD | | .230 | 1.026 | | .092 | | .224 | | .823 | | -1.817 | | 2.276 |
|  |  | TT | | -.078 | .136 | | -.070 | | -.570 | | .571 | | -.349 | | .194 |
|  |  | PD  TCF | | .417  .048 | 1.161  .130 | | .128  -.282 | | .963  .371 | | .039  .002 | | 1.198  .211 | | 3.433  .307 |
| a. Dependent Variable: TIP | | | | | | | | | | | | | | | |
