# Supplementary material for: Centralization or decentralization? Power allocation in team innovation management
Source: PLoS One. 2024 Oct 28;19(10):e0310719. doi: 10.1371/journal.pone.0310719 (PMC11516181; doi:10.1371/journal.pone.0310719)
Supplement: S6 File — (DOCX) [file pone.0310719.s006.docx]

The regression of Model 4 (TCD—PD)

| **Entered／Removed variables^a^** | | | |
| --- | --- | --- | --- |
| Model | Entered variables | Removed variables | Method |
| 1 | PD, TT, TS, GD^b^ | . | Enter |
| a. Dependent Variable: TCD | | | |
| b. All requested variables have been entered. | | | |

| **Model Summary^b^** | | | | | | | | | | | |
| --- | --- | --- | --- | --- | --- | --- | --- | --- | --- | --- | --- |
| Model | R | R Square | Adjusted R Square | Std Error of the Estimate | Change Statistics | | | | | Durbin-Watson |  |
|  |  |  |  |  | R Square  Change | F Change | df1 | df2 | Sig. F Change |  |  |
| 1 | .210^a^ | .044 | -.010 | .53820 | .044 | 8.821 | 4 | 71 | .006 | 1.485 |  |
| a. Predictive Variables: (Constant), PD, TT, TS, GD. | | | | | | | | | | | |
| b. Dependent Variable: TCD | | | | | | | | | | | |

| **Anova^a^** | | | | | | | | | | | | |  |  |  |
| --- | --- | --- | --- | --- | --- | --- | --- | --- | --- | --- | --- | --- | --- | --- | --- |
| Model | | Sum of Squares | | | df | | Mean Square | | F | | Sig. | |  |  |  |
| 1 | Regression | .951 | | | 4 | | .238 | | 8.821 | | .006^b^ | |  |  |  |
|  | Residual | 20.566 | | | 71 | | .290 | |  | |  | |  |  |  |
|  | Total | 21.517 | | | 75 | |  | |  | |  | |  |  |  |
| a. Dependent Variable: TCD | | | | | | | | | | | | |  |  |  |
| b. Predictive Variables: (Constant), PD, TT, TS, GD. | | | | | | | | | | | | |  |  |  |
| **Coefficients^a^** | | | | | | | | | | | | |  |  |  |
| Model | | | | Unstandardized Coefficients | | | standardized Coefficients | | t | | Sig. | | 95.0% CI For B | | |
|  |  |  |  | B | Std. Error | | Beta | |  |  |  |  | Lower Bound | | Upper Bound |
| 1 | | (Constant) | | 4.518 | .394 | |  | | 11.456 | | .000 | | 3.732 | | 5.304 |
|  |  | TS | | -.037 | .029 | | -.053 | | -1.297 | | .199 | | -.094 | | .020 |
|  |  | GD | | -1.187 | .879 | | -.061 | | -1.350 | | .181 | | -2.939 | | .566 |
|  |  | TT | | -.018 | .116 | | -.018 | | -.154 | | .878 | | -.249 | | .213 |
|  |  | PD | | .718 | .996 | | .352 | | .018 | | .986 | | .968 | | 2.004 |
| a. Dependent Variable: TCD | | | | | | | | | | | | | | | |
