# Supplementary material for: Centralization or decentralization? Power allocation in team innovation management
Source: PLoS One. 2024 Oct 28;19(10):e0310719. doi: 10.1371/journal.pone.0310719 (PMC11516181; doi:10.1371/journal.pone.0310719)
Supplement: S7 File — (DOCX) [file pone.0310719.s007.docx]

The regression of Model 5 (TCD—PD+PL)

| **Entered／Removed variables^a^** | | | |
| --- | --- | --- | --- |
| Model | Entered variables | Removed variables | Method |
| 1 | PL, TT, PD, GD, TS^b^ | . | Enter |
| a. Dependent Variable: TCD | | | |
| b. All requested variables have been entered. | | | |

| **Model Summary^b^** | | | | | | | | | | | |
| --- | --- | --- | --- | --- | --- | --- | --- | --- | --- | --- | --- |
| Model | R | R Square | Adjusted R Square | Std Error of the Estimate | Change Statistics | | | | | Durbin-Watson |  |
|  |  |  |  |  | R Square  Change | F Change | df1 | df2 | Sig. F Change |  |  |
| 1 | .431^a^ | .185 | .127 | .50040 | .185 | 3.186 | 5 | 70 | .012 | 1.614 |  |
| a. Predictive Variables: (Constant), PL, TT, PD, GD, TS. | | | | | | | | | | | |
| b. Dependent Variable: TCD | | | | | | | | | | | |

| **Anova^a^** | | | | | | | | | | | | |  |  |  |
| --- | --- | --- | --- | --- | --- | --- | --- | --- | --- | --- | --- | --- | --- | --- | --- |
| Model | | Sum of Squares | | | df | | Mean Square | | F | | Sig. | |  |  |  |
| 1 | Regression | 3.989 | | | 5 | | .798 | | 3.186 | | .012^b^ | |  |  |  |
|  | Residual | 17.528 | | | 70 | | .250 | |  | |  | |  |  |  |
|  | Total | 21.517 | | | 75 | |  | |  | |  | |  |  |  |
| a. Dependent Variable: TCD | | | | | | | | | | | | |  |  |  |
| b. Predictive Variables: (Constant), PL, TT, PD, GD, TS. | | | | | | | | | | | | |  |  |  |
| **Coefficients^a^** | | | | | | | | | | | | |  |  |  |
| Model | | | | Unstandardized Coefficients | | | standardized Coefficients | | t | | Sig. | | 95.0% CI For B | | |
|  |  |  |  | B | Std. Error | | Beta | |  |  |  |  | Lower Bound | | Upper Bound |
| 1 | | (Constant) | | 2.905 | .591 | |  | | 4.917 | | .000 | | 1.727 | | 4.083 |
|  |  | TS | | -.014 | .027 | | -.059 | | -.527 | | .600 | | -.069 | | .040 |
|  |  | GD | | -1.136 | .817 | | -.154 | | -1.389 | | .169 | | -2.765 | | .494 |
|  |  | TT | | -.016 | .108 | | -.016 | | -.149 | | .882 | | -.231 | | .199 |
|  |  | PD  PL | | 1.205  .366 | .928  .105 | | .324  .387 | | -.221  3.483 | | .000  .001 | | -2.056  .157 | | 1.646  .576 |
| a. Dependent Variable: TCD | | | | | | | | | | | | | | | |
