# Supplementary material for: Centralization or decentralization? Power allocation in team innovation management
Source: PLoS One. 2024 Oct 28;19(10):e0310719. doi: 10.1371/journal.pone.0310719 (PMC11516181; doi:10.1371/journal.pone.0310719)
Supplement: S8 File — (DOCX) [file pone.0310719.s008.docx]

The regression of Model 6 (TCD—PD+PL+PD*PL)

| **Entered／Removed variables^a^** | | | |
| --- | --- | --- | --- |
| Model | Entered variables | Removed variables | Method |
| 1 | PDPL, TS, TT, GD, PL, PD^b^ | . | Enter |
| a. Dependent Variable: TCD | | | |
| b. All requested variables have been entered. | | | |

| **Model Summary^b^** | | | | | | | | | | | |
| --- | --- | --- | --- | --- | --- | --- | --- | --- | --- | --- | --- |
| Model | R | R Square | Adjusted R Square | Std Error of the Estimate | Change Statistics | | | | | Durbin-Watson |  |
|  |  |  |  |  | R Square  Change | F Change | df1 | df2 | Sig. F Change |  |  |
| 1 | .431^a^ | .186 | .115 | .50379 | .186 | 2.630 | 6 | 69 | .024 | 1.610 |  |
| a. Predictive Variables: (Constant), PDPL, TS, TT, GD, PL, PD. | | | | | | | | | | | |
| b. Dependent Variable: TCD | | | | | | | | | | | |

| **Anova^a^** | | | | | | | | | | | | |  |  |  |
| --- | --- | --- | --- | --- | --- | --- | --- | --- | --- | --- | --- | --- | --- | --- | --- |
| Model | | Sum of Squares | | | df | | Mean Square | | F | | Sig. | |  |  |  |
| 1 | Regression | 4.005 | | | 6 | | .667 | | 2.630 | | .024^b^ | |  |  |  |
|  | Residual | 17.513 | | | 69 | | .254 | |  | |  | |  |  |  |
|  | Total | 21.517 | | | 75 | |  | |  | |  | |  |  |  |
| a. Dependent Variable: TCD | | | | | | | | | | | | |  |  |  |
| b. Predictive Variables: (Constant), PDPL, TS, TT, GD, PL, PD. | | | | | | | | | | | | |  |  |  |
| **Coefficients^a^** | | | | | | | | | | | | |  |  |  |
| Model | | | | Unstandardized Coefficients | | | standardized Coefficients | | t | | Sig. | | 95.0% CI For B | | |
|  |  |  |  | B | Std. Error | | Beta | |  |  |  |  | Lower Bound | | Upper Bound |
| 1 | | (Constant) | | 2.562 | 1.511 | |  | | 1.696 | | .094 | | -.452 | | 5.575 |
|  |  | TS | | -.015 | .028 | | -.060 | | -.531 | | .597 | | -.070 | | .040 |
|  |  | GD | | -1.097 | .838 | | -.149 | | -1.309 | | .195 | | -2.768 | | .574 |
|  |  | TT | | -.014 | .109 | | -.014 | | -.124 | | .902 | | -.231 | | .204 |
|  |  | PD  PL  PDPL | | 1.373  .447  .383 | 6.459  .343  1.553 | | .164  .472  .212 | | .213  1.304  -.247 | | .002  .007  .006 | | -11.513  -.237  1.481 | | 14.259  1.130  2.714 |
| a. Dependent Variable: TCD | | | | | | | | | | | | | | | |
