# Supplementary material for: Centralization or decentralization? Power allocation in team innovation management
Source: PLoS One. 2024 Oct 28;19(10):e0310719. doi: 10.1371/journal.pone.0310719 (PMC11516181; doi:10.1371/journal.pone.0310719)
Supplement: S9 File — (DOCX) [file pone.0310719.s009.docx]

The regression of Model 7 (TCF—PD)

| **Entered／Removed variables^a^** | | | |
| --- | --- | --- | --- |
| Model | Entered variables | Removed variables | Method |
| 1 | PD, TT, TS, GD^b^ | . | Enter |
| a. Dependent Variable: TCF | | | |
| b. All requested variables have been entered. | | | |

| **Model Summary^b^** | | | | | | | | | | | |
| --- | --- | --- | --- | --- | --- | --- | --- | --- | --- | --- | --- |
| Model | R | R Square | Adjusted R Square | Std Error of the Estimate | Change Statistics | | | | | Durbin-Watson |  |
|  |  |  |  |  | R Square  Change | F Change | df1 | df2 | Sig. F Change |  |  |
| 1 | .130^a^ | .017 | -.038 | .57309 | .017 | 7.305 | 4 | 71 | .004 | 2.171 |  |
| a. Predictive Variables: (Constant), PD, TT, TS, GD. | | | | | | | | | | | |
| b. Dependent Variable: TCF | | | | | | | | | | | |

| **Anova^a^** | | | | | | | | | | | | |  |  |  |
| --- | --- | --- | --- | --- | --- | --- | --- | --- | --- | --- | --- | --- | --- | --- | --- |
| Model | | Sum of Squares | | | df | | Mean Square | | F | | Sig. | |  |  |  |
| 1 | Regression | .401 | | | 4 | | .100 | | 7.305 | | .004^b^ | |  |  |  |
|  | Residual | 23.318 | | | 71 | | .328 | |  | |  | |  |  |  |
|  | Total | 23.719 | | | 75 | |  | |  | |  | |  |  |  |
| a. Dependent Variable: TCF | | | | | | | | | | | | |  |  |  |
| b. Predictive Variables: (Constant), PD, TT, TS, GD. | | | | | | | | | | | | |  |  |  |
| **Coefficients^a^** | | | | | | | | | | | | |  |  |  |
| Model | | | | Unstandardized Coefficients | | | standardized Coefficients | | t | | Sig. | | 95.0% CI For B | | |
|  |  |  |  | B | Std. Error | | Beta | |  |  |  |  | Lower Bound | | Upper Bound |
| 1 | | (Constant) | | 3.167 | .420 | |  | | 7.543 | | .000 | | 2.330 | | 4.005 |
|  |  | TS | | .003 | .031 | | .011 | | .091 | | .928 | | -.058 | | .064 |
|  |  | GD | | -.484 | .936 | | -.062 | | -.517 | | .607 | | -2.350 | | 1.382 |
|  |  | TT | | -.124 | .123 | | -.119 | | -1.001 | | .320 | | -.370 | | .123 |
|  |  | PD | | .184 | 1.061 | | .210 | | -.079 | | .007 | | 1.199 | | 2.031 |
| a. Dependent Variable: TCF | | | | | | | | | | | | | | | |
