# Supplementary material for: Centralization or decentralization? Power allocation in team innovation management
Source: PLoS One. 2024 Oct 28;19(10):e0310719. doi: 10.1371/journal.pone.0310719 (PMC11516181; doi:10.1371/journal.pone.0310719)
Supplement: S10 File — (DOCX) [file pone.0310719.s010.docx]

The regression of Model 8 (TCF—PD+PL)

| **Entered／Removed variables^a^** | | | |
| --- | --- | --- | --- |
| Model | Entered variables | Removed variables | Method |
| 1 | PL, TT, PD, GD, TS^b^ | . | Enter |
| a. Dependent Variable: TCF | | | |
| b. All requested variables have been entered. | | | |

| **Model Summary^b^** | | | | | | | | | | | |
| --- | --- | --- | --- | --- | --- | --- | --- | --- | --- | --- | --- |
| Model | R | R Square | Adjusted R Square | Std Error of the Estimate | Change Statistics | | | | | Durbin-Watson |  |
|  |  |  |  |  | R Square  Change | F Change | df1 | df2 | Sig. F Change |  |  |
| 1 | .138^a^ | .019 | -.051 | .57650 | .019 | 7.273 | 5 | 70 | .026 | 2.176 |  |
| a. Predictive Variables: (Constant), PL, TT, PD, GD, TS. | | | | | | | | | | | |
| b. Dependent Variable: TCF | | | | | | | | | | | |

| **Anova^a^** | | | | | | | | | | | | |  |  |  |
| --- | --- | --- | --- | --- | --- | --- | --- | --- | --- | --- | --- | --- | --- | --- | --- |
| Model | | Sum of Squares | | | df | | Mean Square | | F | | Sig. | |  |  |  |
| 1 | Regression | .454 | | | 5 | | .091 | | 7.273 | | .026^b^ | |  |  |  |
|  | Residual | 23.265 | | | 70 | | .332 | |  | |  | |  |  |  |
|  | Total | 23.719 | | | 75 | |  | |  | |  | |  |  |  |
| a. Dependent Variable: TCF | | | | | | | | | | | | |  |  |  |
| b. Predictive Variables: (Constant), PL, TT, PD, GD, TS. | | | | | | | | | | | | |  |  |  |
| **Coefficients^a^** | | | | | | | | | | | | |  |  |  |
| Model | | | | Unstandardized Coefficients | | | standardized Coefficients | | t | | Sig. | | 95.0% CI For B | | |
|  |  |  |  | B | Std. Error | | Beta | |  |  |  |  | Lower Bound | | Upper Bound |
| 1 | | (Constant) | | 2.953 | .681 | |  | | 4.340 | | .000 | | 1.596 | | 4.311 |
|  |  | TS | | .006 | .032 | | .023 | | .183 | | .855 | | -.057 | | .069 |
|  |  | GD | | -.477 | .942 | | -.062 | | -.507 | | .614 | | -2.355 | | 1.401 |
|  |  | TT | | -.123 | .124 | | -.119 | | -.993 | | .324 | | -.371 | | .124 |
|  |  | PD  PL | | .114  .249 | 1.069  .121 | | .273  .249 | | -.106  .401 | | .006  .000 | | 1.247  .093 | | 2.019  .290 |
| a. Dependent Variable: TCF | | | | | | | | | | | | | | | |
