# Supplementary material for: Centralization or decentralization? Power allocation in team innovation management
Source: PLoS One. 2024 Oct 28;19(10):e0310719. doi: 10.1371/journal.pone.0310719 (PMC11516181; doi:10.1371/journal.pone.0310719)
Supplement: S11 File — (DOCX) [file pone.0310719.s011.docx]

The regression of Model 9 (TCF—PD+PL+PD*PL)

| **Entered／Removed variables^a^** | | | |
| --- | --- | --- | --- |
| Model | Entered variables | Removed variables | Method |
| 1 | PDPL, TS, TT, GD, PL, PD^b^ | . | Enter |
| a. Dependent Variable: TCF | | | |
| b. All requested variables have been entered. | | | |

| **Model Summary^b^** | | | | | | | | | | | |
| --- | --- | --- | --- | --- | --- | --- | --- | --- | --- | --- | --- |
| Model | R | R Square | Adjusted R Square | Std Error of the Estimate | Change Statistics | | | | | Durbin-Watson |  |
|  |  |  |  |  | R Square  Change | F Change | df1 | df2 | Sig. F Change |  |  |
| 1 | .140^a^ | .020 | -.066 | .58050 | .020 | 2.231 | 6 | 69 | .005 | 2.172 |  |
| a. Predictive Variables: (Constant), PDPL, TS, TT, GD, PL, PD. | | | | | | | | | | | |
| b. Dependent Variable: TCF | | | | | | | | | | | |

| **Anova^a^** | | | | | | | | | | | | |  |  |  |
| --- | --- | --- | --- | --- | --- | --- | --- | --- | --- | --- | --- | --- | --- | --- | --- |
| Model | | Sum of Squares | | | df | | Mean Square | | F | | Sig. | |  |  |  |
| 1 | Regression | .467 | | | 6 | | .078 | | 2.231 | | .005^b^ | |  |  |  |
|  | Residual | 23.252 | | | 69 | | .337 | |  | |  | |  |  |  |
|  | Total | 23.719 | | | 75 | |  | |  | |  | |  |  |  |
| a. Dependent Variable: TCF | | | | | | | | | | | | |  |  |  |
| b. Predictive Variables: (Constant), PDPL, TS, TT, GD, PL, PD. | | | | | | | | | | | | |  |  |  |
| **Coefficients^a^** | | | | | | | | | | | | |  |  |  |
| Model | | | | Unstandardized Coefficients | | | standardized Coefficients | | t | | Sig. | | 95.0% CI For B | | |
|  |  |  |  | B | Std. Error | | Beta | |  |  |  |  | Lower Bound | | Upper Bound |
| 1 | | (Constant) | | 3.268 | 1.741 | |  | | 1.877 | | .065 | | -.205 | | 6.740 |
|  |  | TS | | .006 | .032 | | .023 | | .188 | | .851 | | -.058 | | .070 |
|  |  | GD | | -.512 | .965 | | -.066 | | -.531 | | .597 | | -2.438 | | 1.413 |
|  |  | TT | | -.126 | .126 | | -.121 | | -1.001 | | .321 | | -.376 | | .125 |
|  |  | PD  PL  PDPL | | 1.561  .025  -.351 | 7.443  .395  1.789 | | .177  .225  -.185 | | -.210  -.064  .196 | | .005  .000  .005 | | 10.409  .113  -3.217 | | 13.288  .762  -1.920 |
| a. Dependent Variable: TCF | | | | | | | | | | | | | | | |
